# Association of antimicrobial consumption with *Clostridioides difficile* incidence across the departments of an academic medical centre

**DOI:** 10.1016/j.infpip.2025.100468

**Published:** 2025-05-19

**Authors:** Nasstasja Wassilew, Alexandra Zehnder, Andrew Atkinson, Andreas Kronenberg, Jonas Marschall

**Affiliations:** a Department of Infectious Diseases and Hospital Epidemiology, Bern University Hospital, University of Bern, Bern, Switzerland; b Institute for Infectious Diseases, University of Bern, Bern, Switzerland; c Division of Infectious Diseases, Washington University School of Medicine, St. Louis, MO, US; d Division of Infectious Diseases, University of Arizona, College of Medicine, Phoenix, AZ, US

**Keywords:** *Clostridioides difficile*, Incidence, Antibiotic consumption, Correlation, Acute care hospital, Nosocomial

## Abstract

**Background:**

*Clostridioides difficile* infection *(CDI)* is a common gastrointestinal disease in healthcare settings, ranging from uncomplicated diarrhoea to life-threatening pseudomembranous colitis. It is associated with increased morbidity, mortality and healthcare costs. The aim of the study was to correlate CDI incidence with total and specific antibiotic consumption across 17 clinical departments of an academic hospital.

**Methods:**

This retrospective correlation study used data on CDI and antibiotic prescriptions from 1.1.2008 to 31.12.2021. CDI episodes were defined using CDC criteria. Antibiotic consumption was reported per WHO in defined daily doses (DDD). A mixed effects logistic regression model was fitted with each department as random effect to determine CDI incidence as a function of year and adjusted for antibiotic consumption.

**Results:**

Amoxicillin-clavulanate showed the highest annual consumption across the 17 departments (median 13.5 DDD/100 patient-days). The average CDI incidence was highest in nephrology (22.3/10′000 patient-days) and lowest in otorhinolaryngology (0.1/10′000 patient-days). We observed an association between overall antimicrobial consumption and CDI incidence (incidence risk ratio (IRR) per 10 DDD/100 patient-days of 1.16, 95% confidence interval (1.09, 1.23), *P*<0.001). When plotting each department's CDI incidence against the departmental average annual consumption, no significant trend was found; however, there was a trend for the association between CDI and selected antibiotic usage, such as carbapenems (*P*=0.003), ceftriaxone (*P*=0.04), cefepime (*P*<0.001), macrolides (*P*<0.001) and piperacillin/tazobactam (*P*=0.03).

**Conclusions:**

We detected an association between antibiotic consumption and CDI incidence across the departments of an academic hospital; however, we could only correlate departmental CDI incidence with the usage of select antibiotics.

## Introduction

*Clostridioides difficile (C. difficile)*, a spore forming Gram-positive anaerobic bacillus, is a common cause of gastrointestinal infection in healthcare settings [1]. The spectrum of *Clostridioides difficile* infection (CDI) ranges from uncomplicated diarrhoea to life-threatening pseudomembranous colitis, and is associated with increased morbidity, mortality and healthcare costs [2,3]. Aside from predisposing factors such as host characteristics (advanced age, comorbidities), poor infection control practices [4], and the use of gastric acid-suppressive agents [5], the main trigger for CDI is antibiotic exposure, by means of disrupting the normal intestinal flora. Although the association between antibiotic consumption and the risk for developing CDI is well established, the risk profile of antibiotic groups or individual antimicrobial agents is insufficiently characterized [[6], [7], [8]]. This information could guide antibiotic stewardship interventions [9]. Moreover, we are not aware of any study elucidating the link between antibiotic exposure and CDI risk across individual clinical departments of an acute care hospital.

The objective of this study was to correlate CDI incidence with the use of specific antibiotics or antibiotic groups in different clinical departments of a single academic hospital. We hypothesized that the results could highlight the risks of indiscriminate antibiotic prescribing and guide optimization of prescribing practice, particularly in those departments with a high incidence of CDI.

## Methods

### Study design and hospital setting

We conducted a retrospective correlation study at Bern University Hospital, an academic tertiary care centre with approximately 44,000 admissions per year, resulting in 2.9 million patient-days considered in our analysis. The 17 clinical departments included in the study are listed in the results. We excluded ophthalmology as patients are rarely exposed to systemic antibiotics and CDI occurred only sporadically. Given that infants and children are significantly more likely to be asymptomatic gastrointestinal carriers of *C. difficile* than adults, we excluded the local children's hospital from the analysis [10]. Our study focused on hospitalised patients, patients seen in the emergency department and outpatient clinics were not considered. Throughout the study period, the infection prevention strategy was to start contact precautions once a patient was diagnosed with CDI. At the time, there were no other measures taken to reduce CDI cases, especially no automated process for testing.

### Data collection and study period

Pertinent data were extracted from the National Centre for Antibiotic Resistance, ANRESIS, a national surveillance program that collects data on antibiotic resistance and antibiotic consumption from a majority of Swiss hospitals, including Bern University Hospital [11]. We extracted data for *C. difficile* positive native stool specimens from routine clinical diagnostics performed at Bern University Hospital between 01.01.2008 and 31.12.2021. One significant change in the diagnostic approach took place at Bern University Hospital during the study period: Before December 2010, a toxin A/B enzyme immunoassay was used for the analysis of stool samples, with three stool samples being collected from each patient to increase test sensitivity. In December 2010 this assay was replaced by a two-step diagnostic approach consisting of first: screening for *C. difficile* by a Glutamate dehydrogenase (GDH) enzyme-linked immunosorbent assay (ELISA) (Liaison© *C. difficile* GDH, DiaSorin, Saluggia, Italy), and second: confirmation of Toxin B production by a PCR-based test (GeneXpert©, Cepheid, Sunnyvale CA, US). Since then, only one stool sample per patient was collected and both tests had to be positive to be reported out as *C. difficile*.

### Classification of CDI episodes

*C. difficile* positive isolates were classified according to the Centers for Disease Control and Prevention (CDC) definition [12]. All isolates of each individual patient were categorized into four different groups: 1) first infection, defined as first CDI episode identified at the study hospital, 2) duplicate episode, reflecting another positive CD test <14 days after the first infection within the study period, 3) recurrent episode, defined as a new positive CD test 14–56 days after the first infection within the study period, and 4) new infection, if another detection occurs >56 days after the initial episode.

Duplicate and recurrent episodes were excluded from the analysis. First and new infections were each classified as CDI episodes and considered for this analysis. The incidence of CDI was calculated as CDI episodes per 10,000 patient-days and calendar year. Annual patient-days per department were obtained from the finance and controlling department of Bern University Hospital. The admission day and subsequent hospitalization days counted towards the length of hospital stay but not the day of discharge, according to the OECD definition for bed-days (corresponding to patient-days) [13], which was chosen for comparability.

### Determining antibiotic consumption

Data on antibiotic consumption available in ANRESIS were provided by the Bern University Hospital pharmacy as number of packages delivered to each department from 2008 to 2021, identified by the respective departmental accounting unit. All departments use the same delivery system without keeping significant local antibiotic stocks and without moving stocks from one department to another. Data from the ANRESIS database were aggregated into defined daily doses (DDD) using the ATC/DDD system promoted by the World Health Organization (WHO) [14]. The DDD is the assumed average maintenance dose per day for an antimicrobial agent used for its main indication in adults. Antibiotic consumption in DDD/100 patient-days was calculated per annum for each of the departments included in the analysis. The most frequently prescribed individual antimicrobial agents and antibiotic groups were analysed as follows (Table I): Amoxicillin-clavulanic acid, piperacillin-tazobactam, cefuroxime, ceftriaxone, cefepime, trimethoprim-sulfamethoxazole, doxycycline and clindamycin (each analysed individually). The following antibiotics are presented as antibiotic group: carbapenems, aminoglycosides, fluoroquinolones, and macrolides (considering azithromycin and clarithromycin only). No distinction was made between therapeutic or prophylactic antibiotic use.

**Table I:** Annual consumption of selected antibiotics or antibiotic groups (DDD/100 patient-days) summarized across 17 clinical departments for the years 2008–2021

Antibiotics/Antibiotic group	Median	IQR	Range
Aminoglycoside	0.2	0.0–0.6	(0.0, 25.78)
Carbapenem	1.0	0.3–2.3	(0.0, 7.9)
Cefepime	1.5	0.7–2.8	(0.1, 18.8)
Ceftriaxone	3.0	1.6–5.1	(0.0, 12.8)
Cefuroxime	1.9	0.8–7.4	(0.0, 51.6)
Clindamycin	0.9	0.4–1.9	(0.0, 10.3)
Doxycycline	0.8	0.4–1.3	(0.0, 3.7)
Fluoroquinolone	2.2	1.5–3.6	(0.2, 16.7)
Amoxicillin-clavulanic acid	13.5	7.6–21.5	(3.3, 96.6)
Clarithromycin/azithromycin	0.8	0.3–1.9	(0.0, 13.2)
Piperacillin-tazobactam	1.1	0.5–2.8	(0.0, 14.0)
Trimethoprim sulfamethoxazole	2.0	1.0–3.6	(0.1, 15.7)

Note. DDD=defined daily dose.

### Statistical analysis

Annual antibiotic consumption was defined as median DDD per 100 patient-days, per antibiotic (group), along with the associated interquartile range (IQR), and minimum and maximum values. CDI incidence per 10,000 patient-days was summarized for each hospital department and antibiotic (group) along with 95% confidence intervals.

We plotted both the annual crude CDI incidence and total antibiotic consumption per hospital department over the study period to identify general temporal trends. To further investigate these trends, we fitted uni- and multivariable Poisson models with the dependent variable being the number of CDI cases and as denominator the log_10_ of the number of patient-days, adjusted for antibiotic consumption, year and department. Forwards selection and backwards deletion was used to identify the most parsimonious multivariable model; variables significant at a 10% level or less in univariable models were considered. All statistical analysis were performed in R Version 3.6.1 (R Foundation for Statistical Computing, Vienna, 2017), with a *P*-value of 0.05 considered statistically significant.

## Results

### Antibiotic consumption

There were 951,955 admissions over the study period with median length of stay 4.6 days (interquartile range [2.9, 6.1]). The annual consumptions of the selected antibiotics and antibiotic groups in the hospital are summarised in Table I. Amoxicillin-clavulanic acid was the antibiotic with by far the highest total annual consumption [median of 13.5 DDD/100 patient-days per year across the 17 clinical departments (IQR 7.6–21.5 DDD/100 patient-days; range 3.3–96.6)], followed by ceftriaxone (median 3 DDD/100 patient-days; IQR 1.6–5.1; range 0.0–12.8), and fluoroquinolone antibiotics (median 2.2 DDD/100 patient-days; IQR 1.5–3.6; range 0.2–16.7).

**Figure 1:**
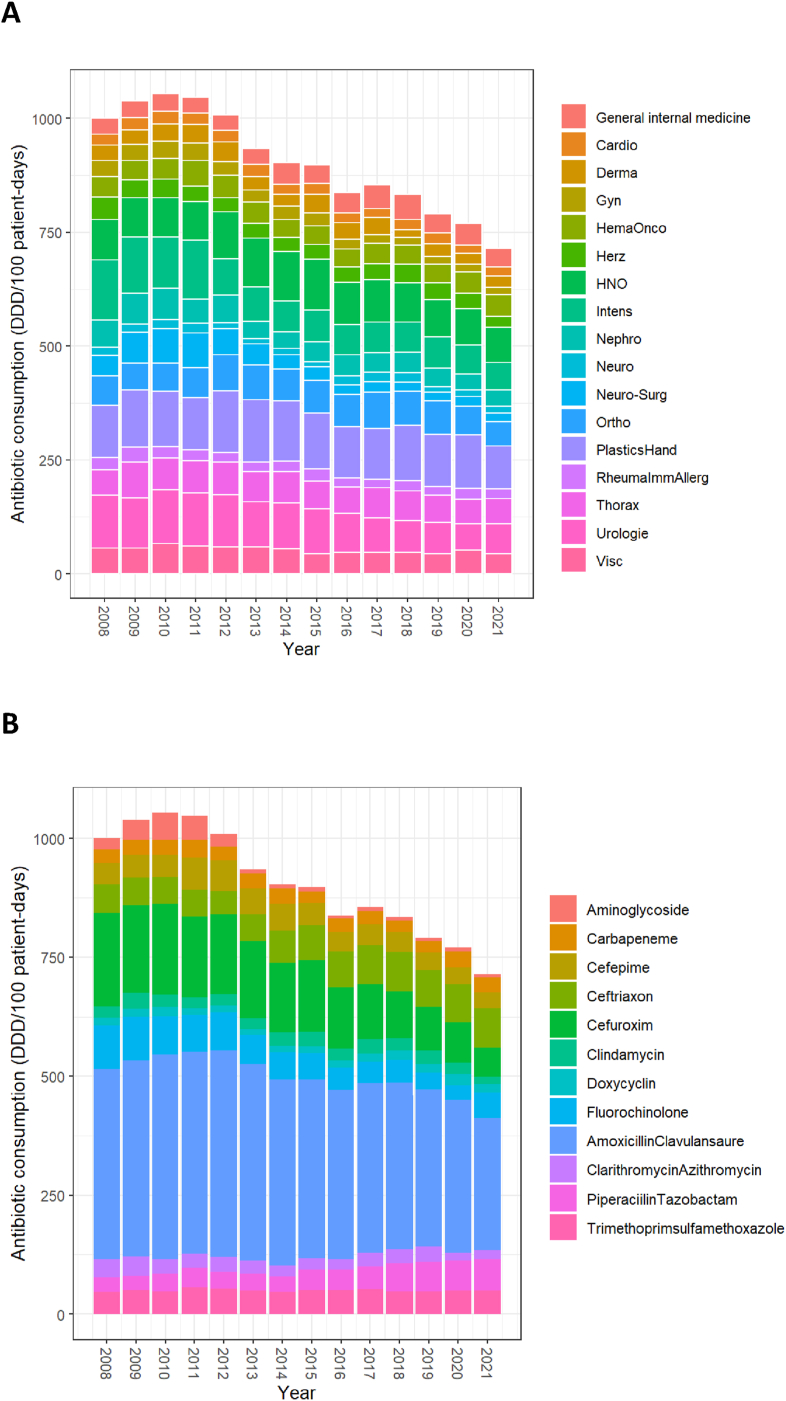
**A:** Overall antibiotic consumption of the studied antibiotics and antibiotic groups, stratified by clinical department and year. For abbreviations, see Table 2. **B:** Overall antibiotic consumption per year, stratified by antibiotic or antibiotic group (total consumption of study antibiotics and antibiotic groups, which are frequently used and/or expected to have an impact on the risk for developing CDI). Footnote. The figures reflect absolute use.

The annual use of the antibiotics examined here varied widely between the 17 clinical departments, with the highest consumption in plastic & hand surgery (weighed median 116.2 DDD/100 patient-days; IQR: 113.8–123.1), urology (weighed median 98.8 DDD/100 patient days; IQR: 70–112.6), and ENT (weighed median 86.4 DDD/100 patient-days; IQR: 84.3, 94.0). The lowest consumption was found in neurology (weighed median 14.6 DDD/100 patient-days; IQR: 11.7–19) (Table II). The total consumption of the study antibiotics and antibiotic groups decreased over the years (Figure 1A, Figure 1B); while consumption remained stable in some departments or dropped somewhat in others, the decrease was most pronounced in intensive care, urology, and neurosurgery (Figure S1). The proportional use of ceftriaxone and piperacillin/tazobactam increased over the 14 years of observation, whereas relative consumption of cefuroxime and fluoroquinolones decreased (Figure 1B).

**Table II:** Average antibiotic consumption per clinical department, 2008–21, and CDI incidence (all years)

Clinical department (abbreviation in parenthesis)	Weighted^a^ median consumption (DDD/100 patient-days) per year [IQR]	CDI incidence per 10′000 patient-days (95% CI)
General internal medicine (AIM)	39.2 [35.3, 44.4]	7.6 (6.8, 8.4)
Cardiology (Cardio)	23.2 [20.2, 25.3]	1.6 (1.1, 2.3)
Dermatology (Derma)	34.2 [28.7, 38.5]	1.2 (0.4, 2.7)
Gynaecology and Obstetrics (Gyn)	23.6 [16.9, 29.8]	1.0 (0.7, 1.4)
Hematology-Oncology HemaOnco)	43.9 [40.8, 46.9]	14.2 (12.7, 16.0)
Cardiovascular surgery (Herz)	33.5 [31.8, 37.4]	6.9 (5.8, 8.0)
Otorhinolaryngology and oral & maxillo-facial surgery (HNO)	86.4 [84.3, 94.0]	0.1 (0.0, 0.5)
Intensive Care (Intens)	67.7 [65.1, 71.0]	10.3 (8.8, 12.0)
Nephrology (Nephro)	44.3 [36.5, 57.4]	22.3 (19.1, 25.8)
Neurology (Neuro)	14.6 [11.7, 19.0]	2.0 (1.4, 2.8)
Neurosurgery (Neuro-Surg)	28.3 [20.5, 51.1]	2.7 (1.9, 3.7)
Orthopedics (Ortho)	69.0 [62.8, 73.2]	4.0 (3.2, 4.9)
Plastics and Hand Surgery (PlasticsHand)	116.2 [113.8, 123.1]	2.8 (1.6, 4.5)
Rheumatology, Immunology and Allergology (RheumImmAllerg)	22.9 [20.2, 24.5]	3.2 (2.0, 4.9)
Thoracic Surgery and Pneumology (Thorax)	63.9 [56.8, 68.6]	17.7 (15.1, 20.5)
Urology (Urologie)	98.8 [70.0, 112.6]	1.7 (1.1, 2.6)
Abdominal surgery and medicine (Visc)	52.6 [45.8, 58.1]	8.3 (7.3, 9.6)

Note. CDI=Clostridioides difficile infection. DDD=Defined daily dose.

a weighted by number of patient-days per year; IQR inter-quartile range [IQR]; CI confidence interval.

### CDI incidence

Of the 2,492 *Clostridioides difficile* positive samples from January 1, 2008 to December 31, 2021 that were analysed, we classified 1,827 as CDI *episodes* (including first episodes and new infections), 137 as CDI recurrences, 353 as CDI duplicates, and 175 other exclusions. Only CDI *episodes* were considered for this analysis.

**Figure 2:**
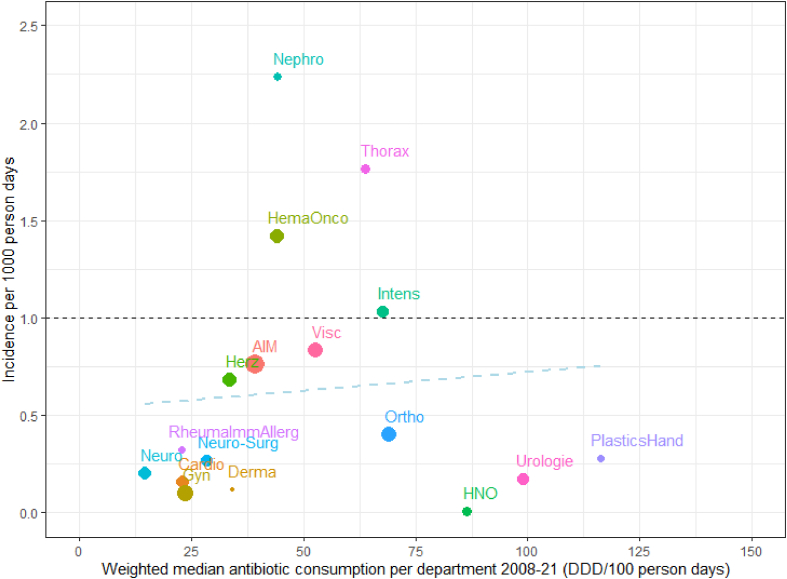
Overall CDI incidence plotted against weighted median yearly antibiotic consumption per 100 patient-days considering all study antibiotics (2008–21); size of bubble is proportional to the number of patient-days; weighted line of best fit shown in light blue, dashed. For abbreviations, see Table II. Footnote. The y axis was re-scaled from incidence per 10′000 as used in the results section to 1000 person days. Person days is equivalent to patient-days. (For interpretation of the references to color in this figure legend, the reader is referred to the Web version of this article.)

Incidence of CDI varied between 5.0 (2021) and 9.8 (2009) episodes/10,000 patient-days, but a decreasing trend could be observed from 2008 to 2021, in line with a slight decrease in antibiotic consumption (Figure S2). There was substantial variability in terms of *C. difficile* incidence between the included clinical departments (Figure 2). The average incidence was highest in nephrology (22.3/10′000 patient-days (95% CI 19.1, 25.8)), pulmonary medicine & thoracic surgery (17.7/10′000 patient-days (95% CI 15.1, 20.5)) and haemato-oncology (14.2/10′000 patient-days (95% CI 12.7, 16.0)); and lowest in otorhinolaryngology and oral & maxillo-facial surgery (each 0.1/10′000 patient-days (95% CI 0.0, 0.5)) (Table II).

### Correlating antibiotic consumption with CDI incidence

When reviewing the total antibiotic consumption over the 14-year study period, a significant correlation between antibiotic consumption and CDI incidence became apparent both in univariate (IRR 1.16 (95% CI 1.09, 1.23; *P*<0.001)) and multivariate analysis (IRR 1.16 (95% CI 1.08, 1.23; *P*<0.001)) (Table III and Table S1).

**Table III:** Estimates from the fitted Poisson model with *Clostridium difficile* infection (CDI) cases as numerator and number of patient-days as denominator (see details on clinical departments in the supplementary data, Table S1)

Dependent variable CDI cases	Univariable			Multivariable (N=237, 17 clinical departments)		
	IRR	95% CI	*P* value	aIRR	95% CI	*P* value
Year	1.00	(0.99, 1.01)	0.7	-	-	NS
Antibiotic consumption (per 10 DDD/100 patient-days steps)	1.16	(1.09, 1.23)	<0.001	1.16	(1.08, 1.23)	<0.001

Legend: IRR incidence risk ratio; aIRR adjusted IRR; CI confidence intervals; nE not estimated in multivariable as non-singular design matrix; NS not significant at the 5% level.

Plotting the overall CDI incidence of each department against the average yearly antibiotic consumption, no significant trend was found due to the high variability between the departments (Figure 2). Nevertheless, several departments with low antibiotic consumption revealed an expected, low incidence of CDI (these were neurology, rheumatology, neurosurgery, cardiology, gynaecology, dermatology, cardiac surgery, general internal medicine and abdominal surgery) (Figure 2). Breaking down the total antibiotic consumption to each of the study antibiotics and antibiotic groups, a marginal trend (*P*-values for slope), suggesting correlation with CDI incidence, was seen for carbapenems (IRR 1.25, 95% CI (1.11, 1.42), *P*=0.003), ceftriaxone (IRR 1.14 (1.02, 1.28), *P*=0.04), cefepime (IRR 1.10 (1.05, 1.15, *P*<0.001), macrolides (IRR 1.24 (1.14, 1.35), *P*<0.001) and piperacillin/tazobactam (IRR 1.09 (1.02, 1.17), *P*=0.03) (Figure S3). The lack of a correlation for fluoroquinolones might be explained by outliers such as urology, a department with high antibiotic consumption but low CDI incidence. The negative trend line for clindamycin might be due to the high variability between the included departments, with, for example, abdominal surgery and general internal medicine using very little clindamycin.

Inspecting the departments with the highest CDI incidence further (these were nephrology, pulmonary medicine & thoracic surgery, and haemato-oncology), there was a visible correlation of CDI incidence with ceftriaxone, fluoroquinolones and trimethoprim/sulfamethoxazole use in nephrology; with ceftriaxone, cefepime, piperacillin/tazobactam, trimethoprim/sulfamethoxazole and macrolide use in pulmonary medicine & thoracic surgery, and with carbapenems, cefepime, and trimethoprim/sulfamethoxazole use in haemato-oncology.

## Discussion

This retrospective study shows a significant association between antibiotic consumption and the incidence of *Clostridioides difficile* infection (CDI) across 17 clinical departments of a single academic tertiary care centre over a period of 14 years. However, the study only found marginal trends suggesting a possible association between CDI risk and certain antibiotics, such as carbapenems, ceftriaxone, cefepime, the macrolides, and piperacillin/tazobactam. There was no association with CDI observed for fluoroquinolones, which is different from what we expected.

The association between broad-spectrum antibiotic consumption and the risk of developing CDI has been described before [6,7,15]. Different study designs were applied to try to extrapolate the most important antibiotics associated with the highest risk for CDI, sometimes adding already known risk factors besides antibiotics, which led to heterogenous results. One single-centre, facility-wide, retrospective, ecologic study evaluated the relationship between antibiotic consumption and the incidence of hospital onset (HO)-CDI, based on a matched-month and also a 1-month delay analysis (for an approximation of causality), first facility-wide and then on a unit level for selected units [16]. The results were heterogeneous, revealing significant associations only for one antibiotic (ceftriaxone) in the matched-month analysis, and for two antibiotic groups (carbapenems, fluoroquinolones) in the 1-month delay analytical approach. Another study examined the effects of antimicrobial consumption, gastric acid-suppressive agent use, and infection control practices on the incidence of CDI in a 426-bed general teaching hospital in Northern Ireland, and looked into a possible temporal relationship between certain groups of antibiotics and CDI incidence [17]. There, statistically significant associations were observed in the case of expanded-spectrum cephalosporins, broad-spectrum cephalosporins, fluoroquinolones, amoxicillin-clavulanic acid, and macrolide antibiotics. Vernaz *et al.* analysed the temporal relationship between antibiotic use and the incidence of CDI by a multivariate transfer function model including lag time, to imply causality between antibiotic use and subsequent CDI. The authors were able to document a statistically significant relationship between the number of both hospital-acquired CDI and all CDI cases and the consumption of piperacillin/tazobactam, ciprofloxacin and cefuroxime [18]. Lastly, a recent ecologic analysis by Kazakova *et al.*, using data from >500 acute care hospitals in the United States, found that for every 10 days of therapy (DOT)/1000 patient-days increase in the use of third- and fourth-generation cephalosporins or carbapenems, there was a corresponding increase of 2.1% and 2.9% in hospital onset (HO-) CDI, respectively [19]. The time series analysis revealed that the six acute care hospitals with a ≥30% decrease in total antibiotic use had a 33% decrease in HO-CDI (rate ratio, 0.67 [95% CI 0.47–0.96]). Additionally, hospitals with a ≥20% decrease in fluoroquinolone or third- and fourth-generation cephalosporin use saw a corresponding decrease in hospital onset CDI by 8% and 13%, respectively.

Interestingly, some departments with elevated antibiotic consumption did not show any correlation between their most frequently prescribed antibiotic and CDI incidence. Urology, for example, consumed large amounts of ceftriaxone, fluoroquinolones, doxycycline, trimethoprim/sulfamethoxazole and amoxicillin/clavulanic acid but had a very low CDI incidence. This might be explained by shorter than average hospitalizations in this department. In other departments with a marked association between high antibiotic consumption and CDI incidence, this might be explained by the effect of additional risk factors, e.g. the use of antineoplastic agents and long courses of antibiotics in the haemato-oncology department, or presumably multi-morbid, elderly patients in nephrology, with kidney disease as key risk factor besides antibiotic exposure [6,20]. Unfortunately, we do not have information on whether some patients developed CDI after discharge from the hospital, as this is not currently tracked in our healthcare system. Taken together, the results presented here suggest that there is a correlation between antibiotic consumptions and CDI incidence across individual clinical departments which, in addition to the literature referenced above, can be used as a reminder for departments to curb their antibiotic use in order to reduce CDI rates. Moreover, this correlation appears to be driven by certain antibiotic agents but not by all.

As a consequence, we believe our findings underline the necessity of an ongoing surveillance of both antibiotic consumption and CDI, encourage us to implement antimicrobial stewardship interventions adapted to local (departmental) antibiotic prescription habits, patient populations and CDI incidences, and suggest that we measure the effect on CDI incidences, as stated by other authors before [9,21].

Our study has a number of strengths: First, this was a retrospective analysis of data generated during routine medical care, and as such was not influenced by any outside factors, so selection and information biases are unlikely. Second, this study captured a large amount of data over an extended period, which is in contrast to most of the earlier studies on the topic. Third, this is to our knowledge the first study to examine antibiotic consumption and CDI incidence across the *clinical departments* of a large academic tertiary care centre.

The study also had several limitations: First, data were only available on an annualized basis, which could have missed correlations between consumption of certain antibiotics and CDI in departments with a high variability of within-department antibiotic consumption. Second, a time-dependent correlation analysis, which tries to account for the delay in CDI occurrence after the start of antibiotic treatment, for example, by correlating antibiotic consumption and CDI detection with a one-month gap, and thus allows for a more specific detection of a CDI-correlated antibiotic “trigger,” was not possible. Third, as we did not collect additional clinical data, we were unable to adjust the CDI risk for individual risk factors other than antibiotic consumption. Fourth, *C. difficile* was a lab-based diagnosis. Even though our lab only processes unformed stools or diarrhoea for *C. difficile,* we were unable to differentiate if a given patient had CDI versus if they had another condition causing diarrhoea and additionally were tested positive for *C. difficile*, being an innocent bystander. Additionally, our lab employs a diagnostic approach based on a PCR method for measuring Toxin B production. This method does not necessarily indicate active toxin production and may lead to an overestimation of true CDI cases. Fifth, incidence could not be plotted to each year and antibiotic, as the graph would have been overloaded and essentially unreadable. Finally, antibiotic consumption was based on delivery of antibiotics rather than actual «consumption» by a patient, and then recalculated on DDD/100 patient-days, which does not necessarily reflect the true amount of antibiotics consumed. Antibiotic consumption based on DOT/100 patient-days reflecting the duration of treatment, rather than a predefined dose of antibiotics would be a more useful metric to measure antibiotic consumption in terms of cumulative risk for an individual patient.

## Conclusion

Indiscriminate use of antibiotics may increase the risk of CDI, and certain antibiotics may be more strongly associated with CDI incidence than others. Here, we report on antibiotic consumption and its association with CDI risk, and the correlation trends we saw for a number of individual antibiotics such as carbapenems (*P*=0.003), ceftriaxone (*P*=0.04), cefepime (*P*<0.001), macrolides (*P*<0.001) and piperacillin/tazobactam (*P*=0.03). The fact that we did not detect an association with CDI in all study antibiotics is likely due to the data being limited to a single academic medical centre - with a larger sample size, it is possible that such associations could have been identified. Antimicrobial Stewardship, by means of promoting the judicious use of antibiotics, is thought to be essential for preventing CDI [9]. Our findings serve as a reminder that the larger the volume of antibiotic consumption is in a given hospital, the greater the risk of *C. difficile* infections. Implementing surveillance programs to highlight this correlation, especially in departments with high incidence as shown in our study (nephrology, pulmonary medicine & thoracic surgery and haemato-oncology), could prepare the ground for interventions aimed at reducing antibiotic consumption and, subsequently, CDI incidence.

## Ethics

Not required.

## Funding

None.

## Conflict of interest

None declared.
