# Supplementary material for: Association of antimicrobial consumption with Clostridioides difficile incidence across the departments of an academic medical centre
Source: Infect Prev Pract. 2025 May 19;7(3):100468. doi: 10.1016/j.infpip.2025.100468 (PMC12268024; doi:10.1016/j.infpip.2025.100468)
Supplement: Multimedia component 1 [file mmc1.docx]

Supplementary data:

Table S1: Estimates from the fitted Poisson model with *Clostridium difficile* infection (CDI) cases as numerator and number of patient-days as denominator.

| Dependent variable  CDI cases | Univariable | | | Multivariable  (N=237, 17 clinical departments) | | |
| --- | --- | --- | --- | --- | --- | --- |
|  | IRR | 95% CI | p value | aIRR | 95% CI | p value |
| Year | 1.00 | (0.99, 1.01) | 0.7 | - | - | NS |
| Antibiotic consumption (per 10 DDD/100 patient-days steps) | 1.16 | (1.09, 1.23) | <0.001 | 1.16 | (1.08, 1.23) | <0.001 |
| Clinical department  General internal medicine  Cardiology  Dermatology  Gynecology and Obstetrics  Hematology-Oncology  Cardiovascular surgery  ORL° and oral and maxillo-facial surgery  Intensive Care  Nephrology  Neurology  Neurosurgery  Orthopedics  Plastics and Hand Surgery  Rheumatology, Immunology and Allergology  Thoracic Surgery and Pneumology  Urology  Abdominal surgery and medicine | 1.00  0.19  0.15  0.13  1.85  0.89  0.01  1.28  2.87  0.25  0.34  0.52  0.34  0.37  2.17  0.21  1.08 | (reference)  (0.06, 0.36)  (0.09, 0.36)  (0.09, 0.19)  (1.58, 2.16)  (0.73-1.07)  (0.00-0.08)  (1.07, 1.55)  (2.40, 3.43)  (0.18, 0.35)  (0.25, 0.48)  (0.41, 0.65)  (0.21, 0.56)  (0.24, 0.58)  (1.81, 2.61)  (0.14, 0.32)  (0.91, 1.28) | <0.001  <0.001  <0.001  <0.001  0.2  <0.001  0.008  <0.001  <0.001  <0.001  <0.001  <0.001  <0.001  <0.001  <0.001  0.4 |  |  | nE |

Legend: °Otorhinolaryngology; IRR incidence risk ratio; aIRR adjusted IRR; CI confidence intervals; nE not estimated in multivariable as non-singular design matrix; NS not significant at the 5% level.

Figure S1: Annual use of the selected study antibiotics in DDD/100 patient-days per clinical department (n=17) from 2008-2021 with weighted median consumption shown dashed. For abbreviations see Table 2.


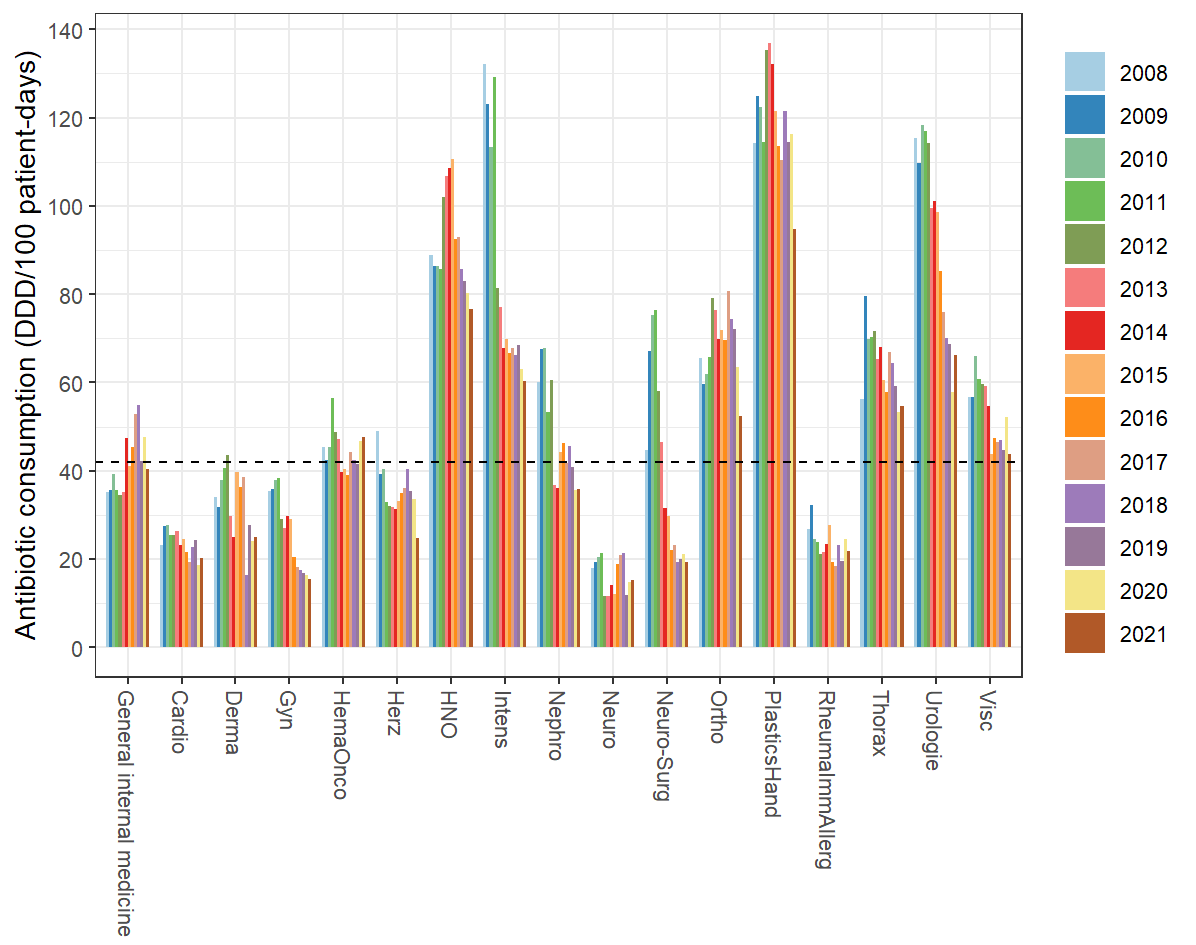


Figure S2: CDI incidence trajectory per 10’000 patient-days 2008-21 (left hand axis), and antibiotic consumption in total DDD/100 patient-days (right hand axis, blue bars); yearly incidence shown as circles with error bars for 95% confidence intervals; line of best fit shown in blue (solid) with 95% confidence interval shaded. The antibiotic consumption of each of the departments is combined into a numerator total.


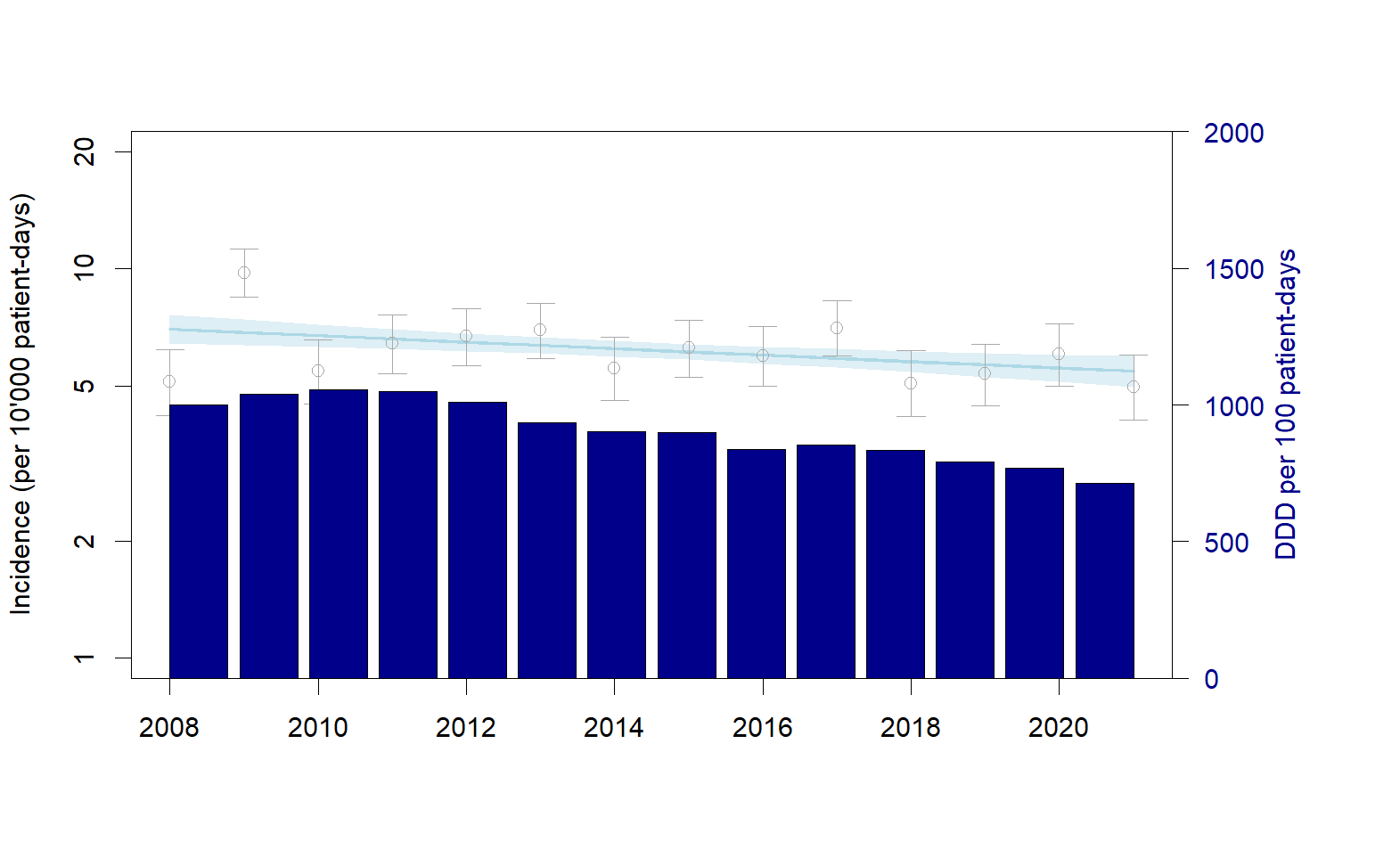


Figure S3: CDI incidence (per 1000 patient-days) and average consumption 2008-21 (DDD/100 patient-days) for each antibiotic (group); size of the bubble is proportional to the number of patient-days; line of best fit (light-blue, solid) with p-value for the IRR top left. Note: Incidence is always the same for a department, so the bubble just moves horizontally in each plot.


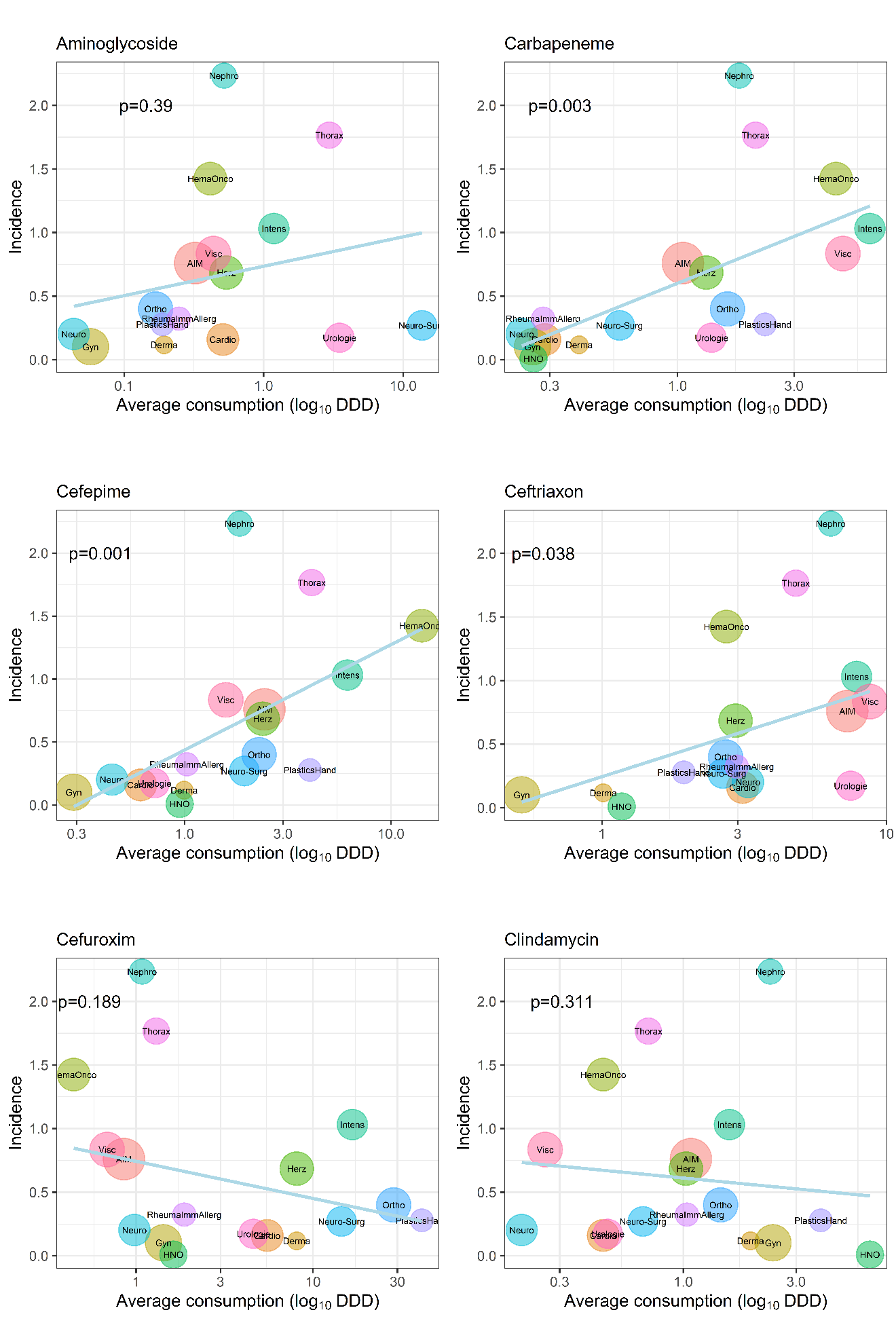


*
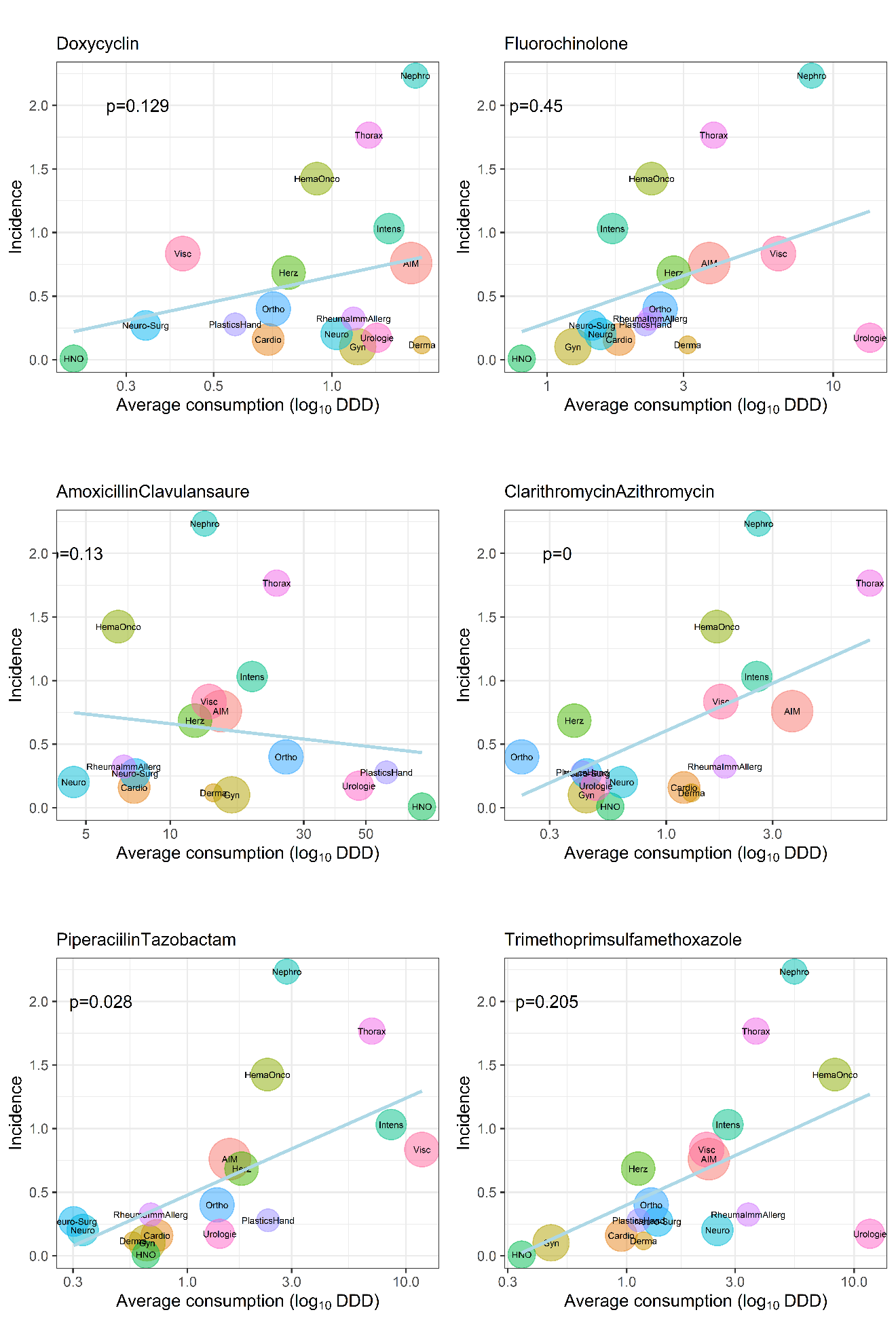
*

*Footnote. For abbreviations, please refer to Table 2.*
